# PSBP-SVM: A Machine Learning-Based Computational Identifier for Predicting Polystyrene Binding Peptides

**DOI:** 10.3389/fbioe.2020.00245

**Published:** 2020-03-31

**Authors:** Chaolu Meng, Yang Hu, Ying Zhang, Fei Guo

**Affiliations:** ^1^College of Intelligence and Computing, Tianjin University, Tianjin, China; ^2^College of Computer and Information Engineering, Inner Mongolia Agricultural University, Hohhot, China; ^3^School of Life Sciences and Technology, Harbin Institute of Technology, Harbin, China; ^4^Department of Pharmacy, Heilongjiang Province Land Reclamation Headquarters General Hospital, Harbin, China

**Keywords:** polystyrene binding peptides, support vector machine, bioinformatic, machine learning, identifier

## Abstract

Polystyrene binding peptides (PSBPs) play a key role in the immobilization process. The correct identification of PSBPs is the first step of all related works. In this paper, we proposed a novel support vector machine-based bioinformatic identification model. This model contains four machine learning steps, including feature extraction, feature selection, model training and optimization. In a five-fold cross validation test, this model achieves 90.38, 84.62, 87.50, and 0.90% SN, SP, ACC, and AUC, respectively. The performance of this model outperforms the state-of-the-art identifier in terms of the SN and ACC with a smaller feature set. Furthermore, we constructed a web server that includes the proposed model, which is freely accessible at http://server.malab.cn/PSBP-SVM/index.jsp.

## Introduction

The immobilization of a biological functional molecule on a solid surface is one of the most important topics in the field of biology. Immobilized enzymes are a typical application of this technology and are commonly used in industrial reactors (Es̨ et al., 2015). The nature of the biocompatibility on the implant surface is considered to be a protein absorption process (Yin et al., 2020). The enzyme-linked immunosorbent assay (ELISA) (Engvall and Perlmann, 1971) is a well-known method for identifying counterparts in biological interactions. This assay is derived from the immobilization target antigen molecules (Li et al., 2018). There are two principles in immobilization: one principle is orienting the target part in the preferred direction, and the other principle is avoiding any unnecessary interaction between the target and the solid surface.

Polystyrene (PS) is used as a protein solid surface in ELISAs and animal cell cultures because of its biological inertia (Kumada et al., 2010). Polystyrene with binding peptides can be used to immobilize bioactive peptides, enzymes and antigens in water at room temperature. These functional monolayer protein layers can be widely applied in the medicine, textile and automobile industries (Yaman et al., 2009; Moritomi et al., 2010; Mrozek and Malysiak-Mrozek, 2011; Modjarrad, 2013). Polystyrene binding peptides (PSBPs) can combine with target proteins or peptides to determine their improper orientation in the immobilization process (Bakhshinejad and Sadeghizadeh, 2016; Wang et al., 2020). The correct recognition of PSBPs is the first and most important step of its related application. It is time-consuming and expensive to use a wet experiment to verify these peptides. To identify PSBPs, we turn to machine learning-based computing strategies. To date, machine learning algorithms have been widely used in biological sequence recognition (Wang et al., 2008, 2010, 2018; Zhou et al., 2017, 2018, 2019; He et al., 2018; Liao et al., 2018; Xu et al., 2018a, b; Bao et al., 2019; Cheng et al., 2019; Ding et al., 2019; Fang et al., 2019; Jin et al., 2019; Liu et al., 2019a; Meng et al., 2019; Shen et al., 2019; Zhu et al., 2019). This process generally includes data collection, feature extraction, feature selection and model training. The positive and negative samples are collected to form a training dataset, and the sequence recognition problem is transformed into a binary classification problem. Discrete features are extracted from training datasets via the feature extraction process. The pseudo amino acid composition (PseAAC) is one of the most commonly used feature extraction algorithms, and many improved algorithms have been produced (Shen and Chou, 2008). The MRMD (Max-Relevance-Max-Distance) (Zou et al., 2018), ANOVA (analysis of variance) (Anderson, 2001) and mRMR (Minimal Redundancy Maximal Relevance) (Ding and Peng, 2005) are commonly used feature selection algorithms. The aim of these feature selection algorithms is overcoming the data redundancy problem. Choosing a good classification algorithm is another particularly important step, and the SVM (support vector machine), random forest, and Bayes classifiers have been widely used to address sequence recognition problems (Li et al., 2019). Ning et al. combined a SVM and the dipeptide composition (DPC) feature, named PSBinder, to construct an identifier to recognize PSBPs (Li N. et al., 2017)^1^. In this study, we used the same training dataset as PSBinder.

In the “Materials and methods” section, we describe the data collection process, the feature extraction method, the ANOVA feature selection, the SVM and the evaluation metrics. We depict the workflow of the proposed identifier and comprehensively analyze the performance of the identifier in the “Results and discussion” section. In the “Conclusion” section, we analyze the shortcomings of the model and look forward to its future improvement.

## Materials and Methods

### Data Collection

We use the same training dataset as PSBinder. This benchmark dataset includes 104 positive samples (PSBPs) and 104 negative samples (non-PSBPs). This dataset is collected from the BDB database (released in January 2017) according to the following criteria. The raw positive samples are selected from nine different phage display libraries. Furthermore, in order to ensure the difference between the positive and negative samples, we attempt to select the same numbers of negative and positive samples from each of the above-mentioned libraries. For those libraries that do not have enough negative samples, we select the same length sequences from the other libraries instead. Then, cysteine amino acids are deleted because they found are at both ends of the circular peptides (Fu et al., 2018). Peptides that contain two specific kinds of characters are removed. One kind is ambiguous characters including “B,” “J,” “O,” “U,” “X,” and “Z.” The other kind is non-alphabetic characters. Two measures are used to screen the above data. Then, we compare each sequence in the positive and negative sample sets, delete the same negative sample sequences and positive sample sequences and replace them with other new negative samples (Yang et al., 2019b). Moreover, the Generalized Jaccard similarity is applied to keep the similarity between the positive and negative samples below 90% (Pan et al., 2009).

### Feature Extraction

The amino acid residue frequency is one of the most important features of protein sequences (Małysiak-Mrozek et al., 2018a; Liu, 2019). The frequency feature can be calculated via the single amino acid composition (AAC), the DPC, three or more peptides’ composition or peptides with a certain gap. There are several proteins or peptide identifiers that have been proposed based on these features. In this paper, we use the weighted frequency of the single AAC and the DPC as the discrete extraction feature.

A peptide consists of 20 kinds of amino acid residues. Thus, a peptide can be presented as follows:

(1) $$p=A_{1}\,A_{2}\,A_{3}\,\ldots \,A_{i}\,\ldots \,A_{L-1}\,A_{L}$$

where *A*_*i*_ is the *i*th amino acid residue of peptide *p* with a length of *L*.

(i) 20-dimensional amino acid composition (AAC)

The weighted frequency of the single AAC is defined as follows:

(2) $${\mathrm{Feature}}_{\mathrm{AAC}}=\{\left(f_{1},f_{2},\ldots ,f_{i},\ldots ,f_{20}\right)|f_{n}=20/420\times (\mathrm{count}\,\left(A_{i}\right)\,\sum_{i=1}^{L}\mathrm{count}\,\left(A_{i}\right)\}$$

where *count*(*A*_*i*_) is the number of *A*_*i*_ in peptide *p*. *Feature*_*AAC*_ consists of 20 vectors, and these vectors represent the weighted frequency of “G,” “A,” “V,” “L,” “I,” “P,” “F,” “Y,” “W,” “S,” “T,” “C,” “M,” “N,” “Q,” “D,” “E,” “K,” “R” and “H.”

(ii) 400-dimensional dipeptide composition (DPC)

The weighted frequency of the DPC is defined as follows:

(3) $${\mathrm{Feature}}_{\mathrm{DPC}}=\{\left(f_{1},f_{2},\ldots ,f_{i},\ldots ,f_{400}\right)|f_{n}=400/420\times (\mathrm{count}\,\left(A_{i}\,A_{j}\right)\,\sum_{i=1}^{L}\mathrm{count}\,\left(A_{i}\,A_{j}\right)\}$$

where *count*(*A*_*i*_*A*_*j*_) represents the number of amino acid residue pairs that consist of *A*_*i*_ and *A*_*j*_. *Feature*_*DPC*_ includes 400 vectors. These vectors represent the weighted frequencies of {“GG,” “GA,”…, “GH,” “AG,” “AA,”…,“HR” and “HH”}.

#### Feature Selection

Generally, the extracted discrete features cannot be directly used in the training of the recognition model because there is noise in them (Yan et al., 2019). Therefore, after feature extraction, we need to use feature selection algorithms to filter the optimal features (Malysiak-Mrozek et al., 2018b). This process is also often considered to be a feature dimensionality reduction process in which noisy features are removed. In this paper, we use ANOVA and the IFS (incremental feature selection) strategy to rank and select the optimal feature set. First, all the extracted features are ranked by their ANOVA scores, and then optimal feature set is selected via incremental feature selection according to a certain criterion (Tang et al., 2019a, b).

(i) ANOVA

The training dataset is composed of positive and negative samples. Thus, each feature can naturally be divided into two groups, that is, the positive group and the negative group. If the difference between the positive and negative groups of a feature is large, then the discriminative ability is good. In ANOVA, the mean square between (MSB) groups and the mean square within (MSW) groups are used to measure the discriminative ability of a feature (Li B. et al., 2017). The MSB groups and the MSW groups of the ξth feature are calculated as follows:

(4) $${\mathrm{MSB}}^{2}\,\left(\xi \right)=\sum_{i=1}^{2}m_{i}\,{\left(\frac{\sum_{j=1}^{m_{i}}{\mathrm{fea}}_{\xi }\,\left(i,j\right)}{m_{i}}-\frac{\sum_{i=1}^{2}\sum_{j=1}^{m_{i}}{\mathrm{fea}}_{\xi }\,\left(i,j\right)}{\sum_{i=1}^{2}m_{i}}\right)}^{2}$$

(5) $${\mathrm{MSW}}^{2}\,\left(\xi \right)=\sum_{i=1}^{2}\sum_{j=1}^{m_{i}}{\left({\mathrm{fea}}_{\xi }\,\left(i,j\right)-\frac{\sum_{j=1}^{m_{i}}{\mathrm{fea}}_{\xi }\,\left(i,j\right)}{m_{i}}\right)}^{2}$$

where *m*_*i*_ is total number of samples in the *i*th group. *fea*_*ξ*_(*i*,*j*) represents the value of the *j*th sample in the *i*th group of the ξth feature. *MSB*^2^(*ξ*) and *MSW*^2^(*ξ*) follow a chi-square distribution with *1* and $\sum_{i=1}^{k}m_{i}-2$ degrees of freedom, respectively.

(6) $${\mathrm{MSB}}^{2}\,\left(\xi \right)∼\chi^{2}\,\left(1\right)$$

(7) $${\mathrm{MSW}}^{2}\,\left(\xi \right)∼\chi^{2}\,\left(\sum_{i=1}^{k}m_{i}-2\right)$$

From eqs 6 and 7, can deduce the following equation:

(8) $$F\left(\xi \right)=\frac{MSB^{2}\left(\xi \right)/1}{MSW^{2}\left(\xi \right)/\sum_{i=1}^{2}m_{i}-2}∼F\left(1,\sum_{i=1}^{2}m_{i}-2\right)$$

*F*(*ξ*) follows an F-distribution with ($1,\sum_{i=1}^{2}m_{i}-2$) degrees of freedom. The larger *F*(*ξ*) is, the greater the contribution of the ξth feature to the classification is.

(ii) Incremental feature selection

All the features are sorted in descending order after calculating eq. 8. The feature sets are generated by adding one new feature at a time as follows:$\left[f\,e\,a_{1}^{{}^{\prime }}\right]$,$\,\left[f\,e\,a_{1}^{{}^{\prime }},f\,e\,a_{2}^{{}^{\prime }}\right]\,\ldots \,\left[f\,e\,a_{1}^{{}^{\prime }},f\,e\,a_{2}^{{}^{\prime }}\,\ldots ,f\,e\,a_{n-1}^{{}^{\prime }}\right]$ and $\left[f\,e\,a_{1}^{{}^{\prime }},f\,e\,a_{2}^{{}^{\prime }}\,\ldots ,f\,e\,a_{n-1}^{{}^{\prime }},f\,e\,a_{n}^{{}^{\prime }}\right]$. The classification models are generated using the above new feature sets, and the best model is selected according to some criteria, such as the accuracy, F1 score or another.

### Support Vector Machine

A support vector machine (SVM) is a kind of generalized linear classifier that classifies data via supervised learning. The SVM maps labeled data to a high-dimensional space and then uses the maximum-margin hyperplane to classify those data. In addition, the SVM is also one of the common kernel learning methods for non-linear classification (Yang et al., 2019a). In recent years, SVMs have been successfully applied in bioinformatics fields (Xiong et al., 2012, 2019; Zhang et al., 2015; Zhang J. et al., 2019; Ding et al., 2016a, b; Wei et al., 2016; Zeng et al., 2017; Zhao et al., 2017; Bu et al., 2018; Xu et al., 2018c; Hu et al., 2019; Liu and Li, 2019; Liu et al., 2019b; Wang et al., 2019; Dou et al., 2020). The LIBSVM is a widely used SVM tool. In addition to the standard SVM algorithm, LIBSVM also includes a support vector regression, multiple classifiers and probability output functions. The source code of LIBSVM is written using C, and it provides a call interface for the mainstream development languages including Java, Python, R and MATLAB. In this paper, the radial basis function (RBF) is used as the kernel function of the SVM. In addition, the grid.py program is used to find the kernel width parameter γ and the penalty constant *C* that optimize the model. In this paper, the search range of $l\,o\,g_{2}^{\gamma }$ is set to [6, 20] and the step size is −0.5. Similarly, the search range of $l\,o\,g_{2}^{C}$is [−10, 20], and the step size is 0.5. We use LIBSVM version 3.24, and it can be downloaded from https://www.csie.ntu.edu.tw/~cjlin/libsvm/.

### Evaluation Measurement

K-fold cross validation, leave-one-out cross-validation (LOOCV) and independent tests are three major validation methods. In this paper, we use five-fold cross validation to evaluate and compare the different identifiers (Jiang et al., 2013; Ding et al., 2017; Wei et al., 2017a,b,c, 2019; Chu et al., 2019; Liu et al., 2019c, d; Shan et al., 2019; Xu et al., 2019c; Zeng et al., 2019a, c; Zhang X. et al., 2019). five-fold cross validation first divides the whole training dataset into five parts. Then, this validation selects four parts to train the model, and the remaining part is used for testing. The above process iterates until all five subsets are used as test datasets. Finally, the five groups of evaluation metric scores are averaged to evaluate the trained model’s performance. To evaluate the model’s performance, we employ the sensitivity (SN), specificity (SP) and accuracy (ACC) to compare the different models. It is worth mentioning that ACC is also used as the objective of model optimization. These evaluation metrics are defined as follows:

(9) $$\mathrm{SN}=\frac{\mathrm{TP}}{\mathrm{TP}+\mathrm{FN}}$$

(10) $$\mathrm{SP}=\frac{\mathrm{TN}}{\mathrm{TN}+\mathrm{FP}}$$

(11) $$\mathrm{ACC}=\frac{\mathrm{TN}+\mathrm{TP}}{\mathrm{TN}+\mathrm{FP}+\mathrm{FN}+\mathrm{TP}}$$

where TN represents true negatives, and TP represents true positives. FN and FP represent false negatives and false positives, respectively.

In addition, the area under the curve (AUC) is also used to evaluate the overall performance of the model. The AUC is the value of the area enclosed by the X, Y coordinates and the receiver operating characteristic curve (ROC curve). The AUC reflects the performance stability of the model. The greater the AUC is, the better the stability of the model.

## Results and Discussion

### The Framework of the Proposed PSBP-SVM Identifier

**FIGURE 1:**
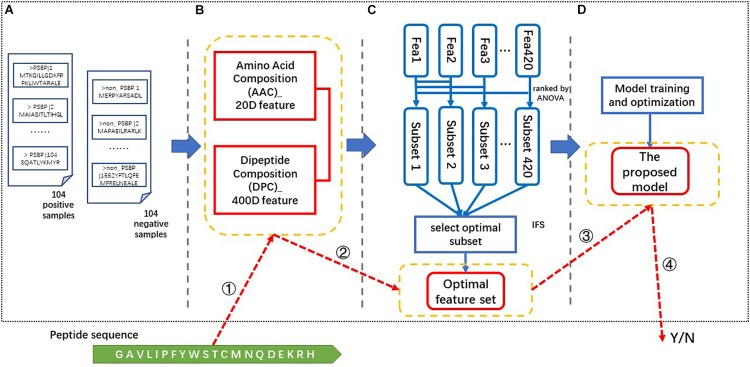
The framework and identification process of the PSBP-SVM. **(A)** Data collection. The benchmark dataset consists of 104 positive samples and 104 negative samples. **(B)** Feature extraction. A 420-dimensional feature is extracted from the benchmark dataset. **(C)** Feature selection. The optimal feature set is generated by the ANOVA ranking algorithm and the IFS process. **(D)** Model training and optimization. The optimal feature set is used to train and optimize the model. The PSBP identification process is based on the four parts in yellow boxes. These parts are ➀ feature extraction, ➁ feature selection, ➂ PSBP identification, and ➃ the result.

There are four steps in the process of constructing our proposed identifier. As illustrated in Figure 1, these steps are data collection, feature extraction, feature selection and model generation and optimization. In the data collection step, the positive and negative samples are collected as described in the “data collection” section. The 420-dimensional AAC and DPC feature is generated from the above benchmark dataset in the feature extraction step. Then, the resulting feature vectors are ranked via their ANOVA scores and a 123-dimensional optimal feature set (123D optimal set) is selected via the IFS process using the ACC as the criterion. This optimal feature set is input into the SVM classifier to train and optimize the model. Finally, the proposed identifier is obtained and called the PSBP-SVM. “PSBP” refers to PSBPs, and the SVM is applied as the classification algorithm.

The identification of a peptide is as follows. ➀The 420-dimensional (AAC + DPC) feature is extracted from this peptide. ➁ Then, we select the feature vectors from the above feature according to the optimal feature set. ➂ ➃Finally, the selected feature vectors are put into the proposed model (PSBP-SVM) to identify whether a peptide is a PSBP or not.

### Comparison With Other Identifiers

To comprehensively investigate the performance of the PSBP-SVM, we compare it with other identifiers including the state-of-the-art identifier. All models presented in this section have been optimized. The optimization conditions of SVM related models are the same as PSBP-SVM.

**FIGURE 2:**
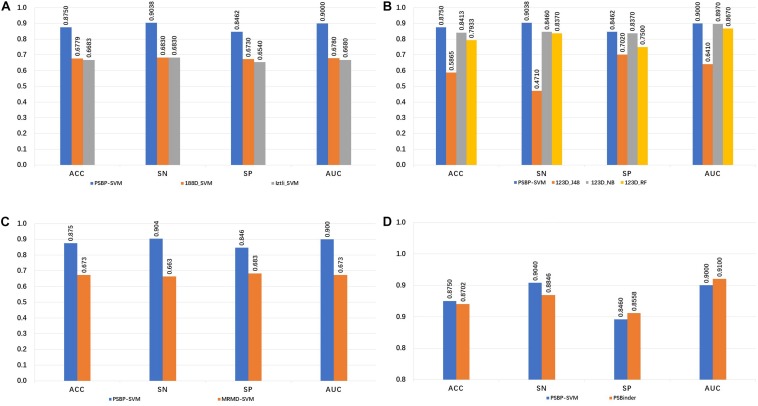
Comparison of the PSBP-SVM and other identifiers. **(A)** Comparison with other feature extraction identifiers. **(B)** Comparison with other classification algorithm identifiers. **(C)** Comparison with other feature selection identifiers. **(D)** Comparison with the state-of-the-art identifier.

The 188-bit (Wei et al., 2018) and Izlti (Diener et al., 2016) feature extraction algorithms are combined with the SVM classifier to generate the 188D_SVM and Iztli_SVM, respectively. The comparison of the PSBP-SVM with the 188D_SVM and Iztli_SVM is illustrated in Figure 2A. In the five-fold cross validation test, the PSBP-SVM achieves 90.38, 84.62, 87.50, and 0.90% SN, SP, ACC, and AUC, respectively. It is observed that the PSBP-SVM is better than the other two identifiers by approximately 20% in terms of the SN, SP, ACC and AUC. This finding demonstrates that the 188-bit and Iztli extraction features might not include important discriminative features of PSBPs and non-PSBPs compared with the 123-dimensional optimal feature set.

To investigate the effectiveness of the SVM classifier, the naive Bayes, random forest and J48 are used to train the identifiers on the 123D optimal set. The generated identifiers of these algorithms are named 123D_NB, 123D_RF and 123D_J48, respectively. From Figure 2B, it is observed that the PSBP-SVM still outperforms the other three identifiers. The performance of 123D_NB follows. The SN, SP, ACC and AUC of 123D_NB are 84.6, 83.7, 84.13, and 0.897%, respectively, which are 5.78, 0.92, 3.37 and 0.003% lower than those of the PSBP-SVM, respectively. 123D_J48 is the worst of all. 123D_J48 only exhibits 47.1, 70.2, 58.65, and 0.641% SN, SP, ACC and AUC, respectively. In particular, the SN of 123D_J48 is below that of random classification. The performance of 123D_RF is worse than that of 123D_NB and better than that of 123D_J48. Thus, it can be concluded that the SVM classifier performs better than other classifier on the 123D optimal feature set.

Different feature selection algorithms lead to different classification effects. Figure 2C represents the influence of two different feature selection algorithms on the model. The MRMD-SVM is generated by replacing part C of Figure 1 with MRMD, that is, MRMD is used as the feature selection algorithm. Finally, a 178-dimensional new optimal feature set is selected by MRMD. From the comparison result, we observe that the MRMD-SVM only achieves 66.3, 68.3, 67.31, and 0.673% in terms of the SN, SP, ACC, and AUC, respectively. The performance of MRMD-SVM is much worse than that of the PSBP-SVM. This result indicates that MRMD may not select important features from the 420-dimensional feature set (Hong et al., 2019).

As shown in Figure 2D, the SN, SP, ACC and AUC values of PSBinder are 88.46, 85.58, 87.02 and 0.91%, respectively, according to the five-fold cross validation test. The SN and ACC of the PSBP-SVM are higher than those of PSBinder by 1.92 and 0.48%, respectively, although the other two metrics are slightly lower. It is worth mentioning that the number of features for the PSBP-SVM is 123, which is smaller than the 146 of PSBinder. Therefore, the PSBP-SVM can effectively avoid overfitting problems compared with PSBinder. For the computing model, the SN value is more significant because it can improve the positive sample identification accuracy by reducing its scope.

### Feature Contribution and Importance Analysis

**FIGURE 3:**
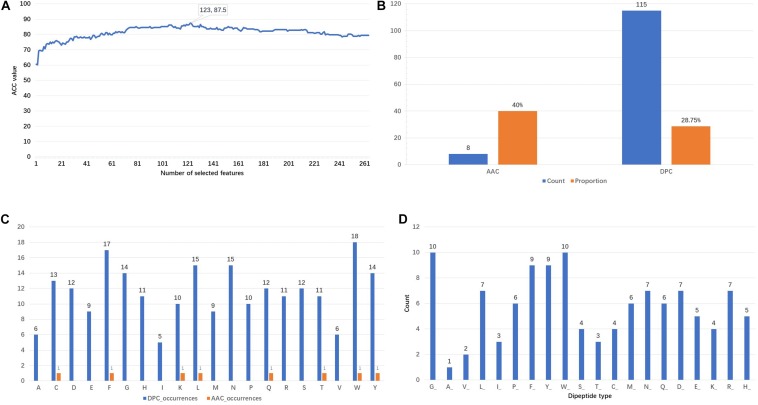
Analysis of the 123D optimal feature set. **(A)** Plot of the accuracy of incremental feature selection. **(B)** Composition of the optimal feature set. **(C)** DPC and AAC occurrences. **(D)** Number of dipeptide types in the DPC.

Figure 3A shows that the ACC values vary with the incremental feature selection process. When the top 123 features are selected, the ACC reaches the highest value of 87.5%. This is also the reason why 123 features are chosen as training features. The analysis of the composition of these optimal features is represented in Figure 3B. It is found that there are 8 AAC features and 115 DPC features, respectively accounting for 40 and 28.75% of the original features. This finding indicates that the AAC features have higher participation rates. Furthermore, the appearance frequencies of 20 amino acids are calculated using the AAC and DPC separately. From the result shown in Figure 3C, we can observe that the top six amino acids both in the AAC and DPC are tryptophan (W), phenylalanine (F), leucine (L), tyrosine (Y), cysteine (C) and glutamine (Q). The counts of the dipeptide types are presented in Figure 3D. The dipeptides that begin with glycine (G), tryptophan (W), phenylalanine (F) and tyrosine (Y) are the top four dipeptide types in the 123D optimal feature set. From the above analysis, we can conclude that tryptophan (W), phenylalanine (F) and tyrosine (Y) play important roles in identifying PSBPs from non-PSBPs.

### Web Server Guidelines

For the convenience of other researchers, we have constructed a web server including the PSBP-SVM, and free access is provided at http://server.malab.cn/PSBP-SVM/index.jsp. This web server includes “Home,” “Dataset,” “About” and “Contact us” pages. One can enter a sequence into the input box of the “Home” page and click the “submit” button to identify whether it is a PSBP or not. Note that only the FASTA format is supported. The “Dataset” page provides a link to download positive and negative samples. The “About” and “Contact us” pages give related information about our proposed model and the authors, respectively.

## Conclusion

In this study, we proposed a novel SVM-based polystyrene binding peptide identification model and incorporated it in an identifier called the PSBP-SVM. The construction process of this model includes feature extraction, feature selection, model training and optimization. The performance comparison shows that the PSBP-SVM outperforms other identifiers, including the state-of-the-art identifier. Furthermore, in order to investigate the contribution of features, we comprehensively analyzed the composition and importance of the optimal feature set used in model training. However, there is still room for improvement in the future. With the help of multiview learning, ensemble learning strategies (Liu and Zhu, 2019; Ru et al., 2019; Zeng et al., 2019b) and evolutionary optimization (Xu et al., 2019a, b), the accuracy can be improved, and the range of the effective features can be further reduced.

## Data Availability Statement

Publicly available datasets were analyzed in this study. This data can be found here: http://server.malab.cn/PSBP-SVM/data.jsp.

## Author Contributions

CM and YH wrote the manuscript, participated in the research design and developed the web server. YZ and FG participated in preparation of the manuscript. CM, YH, FG, and YZ read and approved the final manuscript.

## Conflict of Interest

The authors declare that the research was conducted in the absence of any commercial or financial relationships that could be construed as a potential conflict of interest.

## Footnotes

1 http://i.uestc.edu.cn/sarotup3/cgi-bin/PSBinder.pl
